# Grp78 promotes the invasion of hepatocellular carcinoma

**DOI:** 10.1186/1471-2407-10-20

**Published:** 2010-01-19

**Authors:** Rongjian Su, Zhen Li, Hongdan Li, Huijuan Song, Cuifen Bao, Jia Wei, Liufang Cheng

**Affiliations:** 1 Central Laboratory, Liaoning Medical College, Jinzhou, PR China; 2 Key Lab of Cellular and Molecular Biology, Education Department, Liaoning Province, Jinzhou, PR China; 3 Gastroenterology Department, General Hospital of Chinese Liberation Army, Beijing, PR China

## Abstract

**Background:**

Glucose regulated protein 78 (Grp78) is involved in the invasion and metastasis in many human cancers including gastric cancer, breast cancer, prostate cancer. But the role of Grp78 in the invasion of human hepatocellular carcinoma has not been reported. In this article, we examined if Grp78 was associated with the invasion of hepatocellular carcinoma and explored the possible underlying mechanism.

**Methods:**

The Grp78 and FAK expression levels in 44 patients with hepatocellular carcinoma were examined using immunohistochemistry. Grp78 overexpressing SMMC7721 cells were established by pcDNA3.1 (+)-Grp78 transfection and screened by G418. Grp78 and FAK levels in Grp78 overexpressing cells were down-regulated by siRNA transfection. The invasion status of tumor cells was evaluated by transwell assay in vitro, and chick embryo metastasis model in vivo. Cell spreading was determined by cell spreading assay, and quantitatively measured by Orisis software HUG. Grp78, pY397 FAK, pY576/577 FAK and FAK levels were detected by western blot. RhoA activity was detected by GST pulldown assay. The distribution of actin cytoskeleton was observed by fluorescent staining.

**Results:**

Grp78 expression levels in 44 patients with hepatocellular carcinoma were negatively correlated with tumor grading, and positively correlated with portal invasion and intra-hepatic invasion. Overexpression of Grp78 in SMMC7721 cells promoted the invasion of cancer cells in vitro and in vivo, and this increase in tumor cell invasion was blocked by Grp78 siRNA knockdown. Our results also revealed that overexpression of Grp78 in SMMC7721 cells accelerated the process of cell spreading and promoted lamellipodia formation. Further analysis showed that overexpression of Grp78 in SMMC7721 cells increased pY397 and pY576/577 levels of FAK. Grp78 siRNA knockdown decreased FAK activation and activity. Our results also revealed that Grp78 overexpression in SMMC7721 cells decreased RhoA-GTP level, and Grp78 siRNA knockdown rescued RhoA-GTP level in Grp78 overexpressing cells, indicating Grp78 inhibited RhoA activity in hepatocellular carcinoma cells. Furthermore, overexpression of Grp78 in SMMC7721 cells increased phospho-p190RhoGAP level. FAK siRNA knockdown in Grp78 overexpressing cells reversed phospho-p190RhoGAP level. These data suggested that Grp78 inhibited RhoA activity by up-regulated phospho-p190RhoGAP level and Grp78 mediated p190RhoGAP phosphorylation is FAK dependent.

**Conclusion:**

Grp78 promoted the invasion of hepatocellular carcinoma both in vitro and in vivo. Overexpression of Grp78 in hepatocellular carcinoma cells enhanced the activation and activity of FAK which negatively regulated Rock kinase activity by promoting the phosphorylation of p190RhoGAP.

## Background

Hepatocellular carcinoma (HCC) is the fifth most common cancer of men and the eighth most cancer of women worldwide, which causes 250, 000 deaths each year[1,2]. Early HCC is clinically silent and often well advanced at the first manifestation, only 10-20% of patients are suitable for surgical treatment [2]. Even having undergone surgical treatment, the prognosis of patients with HCC is still poor [3]. The poor prognosis is largely attributed to the invasion at early stage in HCC. Thus there is a substantial need for exploring novel treatments to prevent the invasion of HCC.

The glucose-regulated protein Grp78, a stress-induced endoplasmic reticulum(ER) chaperone, is expressed at basal levels in normal adult organs such as the brain, lung and liver, but is strongly induced in tumors [4]. Overexpression of Grp78 in tumor cells has been linked to the progression of many human cancers including colon cancer, lung cancer, gastric cancer, breast cancer etc [5-8]. Most of the previous studies have been focusing on the roles of Grp78 in anti-apoptosis, chemotherapy resistance in human cancers, but it remains unclear whether Grp78 is involved in the regulation of tumor invasion and metastasis [9].

Recent advances have revealed that Grp78 is associated with the invasion and metastasis of many human cancers. The elevated expression of Grp78 is associated with increased lymphatic node metastasis in gastric cancer [10]. The concentration of Grp78 antibody in serum samples of patients with prostate cancer is positively correlated with the invasion potentiality of cancer cells [11]. The expression level of Grp78 in cancer cells is a biomarker for predicting the invasion and metastasis in breast cancer [12].

Tumor invasion and metastasis is an integrated process that requires the coordinated regulation of various signaling molecules including some kinases and phosphatases. These kinases and phosphatases regulate tumor invasion and metastasis by phosphorylation and dephosphorylation of many signal molecules [13]. The protein tyrosine kinase Focal adhesion kinase (FAK) plays a prominent role in tumor invasion and metastasis [14]. Enhanced FAK signaling promotes the invasion and metastasis of cancer cells. The phosphorylation of Y397 activates FAK and provides docking site for Src kinase which contributes to maximal activity of FAK by phosphorylation at Y576/577 of FAK. Rock kinase acts downstream of FAK and plays critical roles in the invasion of tumor cells. Although basal level of Rock activity is essential for tumor invasion, overactivation of Rock kinase inhibits tumor invasion [15].

In this article, we overexpressed Grp78 in human hepatocellular carcinoma cells SMMC7721 and investigated the effect of Grp78 overexpression on the invasion of HCC and explored the possible underlying mechanism.

## Methods

### Reagents

10-day-old white Leghorn chick embryos were provided by the Experimental Animal Center of Liaoning Medical College. ECM-gel, protease inhibitors, TRITC-conjugated phalloidin, and BCIP/NBT staining solution were purchased from Sigma Corporation. Transwell was obtained from Costar Corporation. Control RNA was obtained from cell signaling Corporation. Fugene HD transfection reagent was obtained from Roche Corporation. TransMessengerTM transfection reagent was purchased from Qiagen Corporation. HRP/Fab Polymer conjugated secondary antibodies were obtained from ZhongShan Company. Rock dominant negative recombinant KDIA and Rhotekin-RBD bound to glutathione-S-transferase sepharose beads were kindly given by Dr Chen Yu-Hua (Developmental Biology Department, China Medical University).

### Antibodies

Grp78, RhoA antibodies were purchased from Santa Cruz Corporation. FAK-pY576/577 was the product of cell signaling Corporation. FAK, FAK-pY397 was obtained from Biosource Corporation. ALP-conjugated secondary antibodies were purchased from cell signaling Corporation.

### Human tissue specimens

All 44 cases tissue samples of patients with HCC were obtained from the Department of Gastroenterology of the General Hospital of Chinese Liberation Army. The Ethics Committee of Liaoning Medical College approved and supervised specimen collection procedures. All the experimental performances related to the tissue samples were in compliance with Helsinki Declaration. We have got the permissions of all the patients before specimen collection. All clinicopathological data were retrospectively collected by reviewing the patients' medical charts. The differentiation extents were re-evaluated by two pathologists according to Edmondson-Steiner grading system. None of the patients has received chemotherapy or irradiation before surgery. Clinicopathological data were summarized and listed in table 1.

**Table 1:** Clinicopathological data and the expressions of Grp78 and FAK of 44 patients with HCC

Number	sex	age	HBsAg	Grade	FAK level	Grp78 level	Portal invasion	Intrahepatic invasion
1.	Male	63	+	Well	1	1	N	N
2.	Male	49	-	Well	1	1	N	N
3.	Male	45	-	Well	1	1	N	N
4.	Male	64	+	Well	1	1	N	N
5.	Male	68	+	Well	1	1	N	N
6.	Female	69	+	Well	2	2	N	N
7.	Male	63	+	Well	1	2	N	N
8.	Female	32	-	Well	3	3	Y	N
9.	Male	42	-	Well	2	2	N	N
10.	Male	50	+	Well	2	3	N	N
11.	Male	55	+	Poorly	3	3	Y	Y
12.	Male	63	+	Poorly	3	3	Y	Y
13.	Male	35	+	Poorly	3	3	Y	Y
14.	Male	69	+	Poorly	2	3	Y	Y
15.	Male	36	-	Poorly	1	1	N	N
16.	Male	60	+	Poorly	2	2	Y	N
17.	Male	48	+	Poorly	2	2	N	Y
18.	Male	49	-	Poorly	3	3	Y	Y
19.	Female	73	+	Poorly	2	3	Y	Y
20.	Male	53	+	Poorly	3	3	Y	Y
21.	Male	59	+	Poorly	2	2	Y	N
22.	Male	66	+	Poorly	3	3	Y	Y
23.	Male	33	-	Poorly	1	1	N	N
24.	Male	37	-	Poorly	3	3	Y	Y
25.	Female	67	+	Poorly	1	1	N	N
26.	Male	60	+	Poorly	2	3	Y	Y
27.	Male	43	+	Poorly	2	2	N	N
28.	Male	42	-	Poorly	3	3	Y	Y
29.	Female	54	-	Poorly	3	3	Y	Y
30.	Female	58	+	Poorly	2	2	N	N
31.	Female	41	-	Poorly	3	3	Y	Y
32.	Male	31	-	Moderately	1	1	N	N
33.	Male	39	-	Moderately	2	2	Y	N
34.	Male	59	+	Moderately	1	1	N	N
35.	Female	55	-	Moderately	3	2	N	Y
36.	Male	32	-	Moderately	1	1	N	N
37.	Female	42	-	Moderately	3	3	N	Y
38.	Male	58	+	Moderately	3	1	N	N
39.	Male	55	+	Moderately	2	2	Y	N
40.	Male	45	-	Moderately	3	3	Y	N
41.	Female	42	-	Moderately	3	2	N	N
42.	Female	51	+	Moderately	3	3	Y	Y
43.	Male	68	+	Moderately	3	3	Y	N
44.	Male	57	+	Moderately	3	3	N	N

### Immunohistochemistry

Immunohistochemistry was performed on the formalin-sixed paraffin sections. Briefly, 5 μm sections were dewaxed, rehydrated and incubated in 0.3% (V/V) hydrogen peroxide in PBS (0.01 M, pH 7.6) for 20 min to inactivate endogenous peroxidase. Antigen was retrieved by high pressure for 2 min in citrate buffer (0.01 M sodium citrate, pH 6.0). The sections were then incubated with 1:100 diluted primary antibodies at 4°C overnight, and then stained with HRP/Fab Polymer conjugated secondary antibodies for 30 min at room temperature. The antibodies were revealed by DAB at room temperature for 1 min. The primary antibodies were replaced by PBS as negative control. All sections were examined and scored independently by two investigators without any knowledge of the clinicopathological data of the patients, at least 5 fields were randomly chosen. Expressions of Grp78, FAK were evaluated according to the ratio of positive cells per specimen and staining intensity. The ratio of positive cells per field was evaluated quantitatively and scored as 0 for staining less than 5%, 1 for staining of 5 to 10%, 2 for staining of 10 to 50%, 3 for staining >50%. Intensity was graded as follows: 1, weak; and 2, strong staining. A total score of 0 to 6 was calculated and the scores were designated as 1(score: 0-1), 2 (2-4), and 3 (5-6).

### Cell culture

Human hepatocellular carcinoma cell line SMMC7721 was kindly given by Dr YH Chen (Developmental Biology Department of China Mediacal University). The cells were propagated in DMEM supplemented with 10% FBS, 2 mM glutamine, 100 U/ml penicillin, 100 μg/ml streptomycin at 37°C, 5% CO_2 _-95% O_2_.

### Transfection

Transfection was performed according to the instruction of Roche's Fugene HD protocol. Briefly cells were cultured to 60-70% confluence in a six-well culture plate and transfected with 4 μg plasmid using 16 μl Fugene HD (1:4 ratios). As control, cells were transfected with vectors under the same condition. Stable transfectants were selected in complete medium containing 400 μg/ml G418 for 2-3 weeks. G418 resistant clones were isolated and cultured in complete medium containing 200 μg/ml G418. The positive clones were identified by western blot. The plasmids used in this experiment were pcDNA3.1 (+)-Grp78 and Rock dominant negative recombinant pCAG-KDIA. pcDNA3.1 (+)-Grp78 recombinant was constructed by inserting a 2 kb fragment of human Grp78 cDNA into HindIII and XhoI site of pcDNA3.1 (+) [16].

### RNA interference

The siRNA sequences against Grp78 and FAK were designed by siRNA finder (Ambion, USA), screened by Ui-Tei K's principles and synthesized by Genechem Corporation (Shanghai, China) [17]. The sequences of sense strands of siRNA duplex were as follows: Grp78: 5'-AGACGCUGGAACUAUUGCUUU-3', FAK: 5'-GAUAGUGGACAGUCACAAAUU-3'. Cells in exponential growth phase were plated in six-well plate (5 × 10^5 ^cells/well), allowed to adhere for 24 h and transfected with siRNA. Transfection of siRNA was performed as Qiagen's TransMessengerTM Handbook. Briefly, the cells were incubated for 4 h with the transfection complex containing 4 μg siRNA. After 4 h, the transfection complex was removed and the cells were incubated in complete growth medium for 72 h. The effect of siRNA transfection was confirmed by western blot.

### Chick Embryo Metastasis Assay AND Alu PCR

The chick embryo metastasis assay was performed as Quigley JP [18]. Briefly, 1 × 10^6 ^tumor cells were suspended in 25 μl serum free medium and inoculated on the chorioallantoic membrane (CAM) of 10-day-old white Leghorn chick embryos. The embryos were incubated in a stationary incubator for 7 days. After which the embryonic lungs and livers were harvested and the status of tumor invasion and metastasis were evaluated by human specific Alu PCR using DNA extracts from chick embryonic lungs and livers. Primers specific for the human Alu were as follows: 5'-ACG CCT GTA ATC CCA GCA CTT-3' (sense), 5'-TCG CCC AGG CTG GAG TGC A-3' (antisense). Each PCR reaction was performed in a final volume of 20 μl containing 60 ng of genomic DNA, 2 mM MgCl_2_, 0.4 μM each primer, 200 μM dNTPs, 0.4 units of Taq polymerase for 30 cycles. PCR cycling conditions were as follows: 95°C for 30 s, 63°C for 30 s and 72°C for 30 s. Chick GAPDH was amplified at the same condition as internal control. The products were separated by 1% agarose gel and evaluated by Chemi-Genius gel imaging and analysis system (UK)

### Cell spreading

Cells were trypsinized, re-plated on fibronectin (10 μg/ml) pre-coated coverslips (105 each well) and allowed to spread for 1 h, 2 h respectively. The status of cell spreading and polarity formation were photographed by inverse microscopy and analyzed by Orisis software HUG. Cell spreading was represented by the mean area (pixels) and cell polarity formation was described as the ratio of long axis/short axis.

### Western blot

Cells were harvested, lysed in RIPA buffer (150 mM NaCl, 1% NP-40, 1% SDS, 1 mM PMSF, 10 ug/ml leupeptin, 1 mM aprotinin, 50 mM Tris-Cl, pH 7.4) for 30 min. The protein concentration was determined by the BCA assay. Cell lysate (50 μg each lane) were separated by 10% SDS-PAGE, transferred to PVDF membrane. The membrane was blocked with 5% non-fat milk for 1 h, incubated with 1:1000 diluted primary antibodies for 3 h, stained with 1:2000 diluted ALP-conjugated secondary antibodies for 30 min at room temperature. Bound antibodies were revealed by BCIP/NBT staining. The levels of interested proteins were analyzed by Chemi-Genius gel imaging and analysis system (UK).

### Fluorescence

Cells were harvested, replated on fibronectin pre-coated coverslips (10 μg/ml). After 24 h, cells were fixed in 3.7% formaldehyde in PBS, permeabilized with 0.1% Triton X-100 in PBS, blocked with PBS containing 1% BSA, incubated with TRITC-conjugated phalloidin for 30 min. The slides were mounted by 95% glycerol and observed by ZEISS A-1 fluorescent microscope (Carl ZEISS, Germany).

### Transwell Assay

Transwell invasion assay was performed as Costar's Transwell procedure. Briefly, Cells were seeded to ECM gel pre-coated, porous upper chamber inserts (5 × 10^4 ^each well) and allowed to invade for 12 h. After 12 h, the inserts were inverted and stained with Hochest33258. The numbers of invaded cells were observed and counted using fluorescent microscope. Three fields were randomly chosen and the numbers of penetrated cells were counted.

### RhoA activity assay

RhoA activity was determined by GST pull-down assay. Briefly, the transfected cells were lysed in NP-40 lysis buffer (150 mM NaCl, 1% NP-40,1 mM PMSF, 10 ug/ml leupeptin, 1 mM aprotinin, 50 mM Tris-Cl, pH 7.4) for 30 min, Cell lysates were centrifuged at 4°C at 14,000 g for 5 min to remove particulate material. The protein concentrations were determined by the BCA assay. Protein extracts (containing 50 μg of protein) were incubated at 4°C for 1 h with an equal volume of Rhotekin-RBD bound to glutathione-S-transferase Sepharose beads on a rotator, centrifuged for 14,000 g for 10 min. The pellets were separated by 10% SDS-PAGE and transferred to PVDF membrane for Western blot analysis using anti-RhoA as primary antibody. The abundance of RhoA was quantified by Chemi-Genius gel imaging and analysis system (UK).

### Statistical analysis

Comparison of the protein levels was performed using one way ANOVA. The correlation analysis was performed by spearman test. A P-value less than 0.05 was considered statistically significant.

## Results

### The expression of Grp78 is positively correlated with portal invasion and intra-hepatic invasion in patients with hepatocellular carcinoma

**Figure 1:**
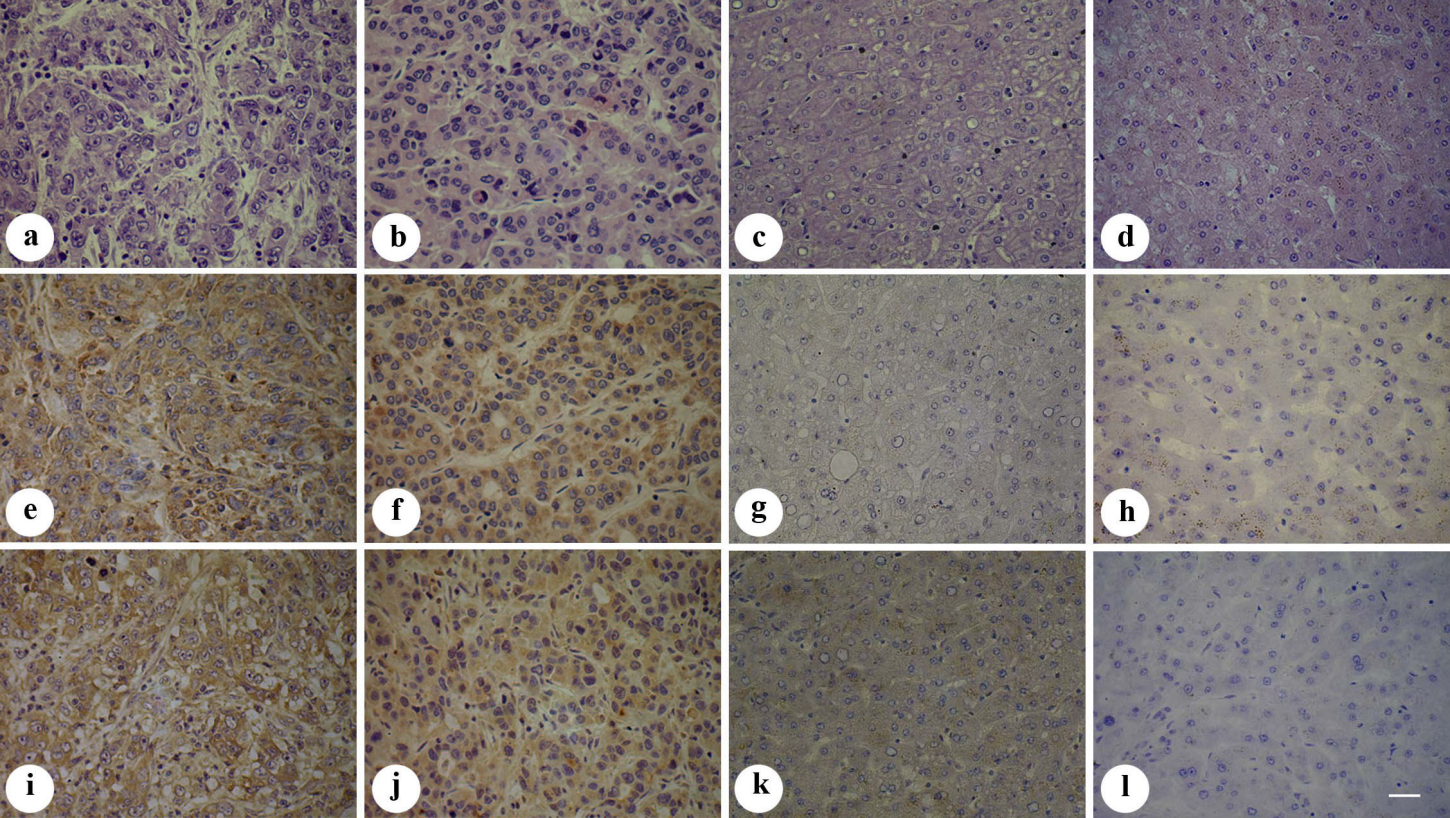
**Immunostaining of Grp78 and FAK in the tissue samples of HCC**. (a-d), HE staining of the tissue samples of HCC. (a), poorly differentiated tissue sample. (b), moderately differentiated tissue sample. (c) Well differentiated tissue sample. (d), peri-cancer tissue sample. (e-l), Grp78 and FAK expressions in poorly differentiated tissue sample (e, i), moderately differentiated tissue sample (f, j), well differentiated tissue sample (g, k) and peri-cancer tissue sample (h, l). (Scale bar = 25 μm).

To investigate Grp78 and FAK expressions in hepatocellular carcinoma, immunohistochemical staining was performed and the results were listed in table 1. As shown in table 1 and table 2, Grp78 and FAK were expressed at high levels in most of poorly differentiated tissue samples (61.9%, 47.6%), and at relatively low levels in most of moderately and well differentiated tissue samples (Fig. 1). Spearman test indicated that Grp78 and FAK expression levels were negatively correlated with the differentiation extent of HCC(r = 0.37, *P *= 0.01; r = 0.312, *P *= 0.04).

**Table 2:** The expression of Grp78 and FAK is negatively correlated with the grade of HCC.

	Grp78					FAK				
Grade	1	2	3	*r*	*P*	1	2	3	*r*	*P*
Well	5(11.4%)	3(6.8%)	2(4.5%)			6(13.6%)	3(6.8%)	1(2.3%)		
Moderately	4(9.1%)	4(9.1%)	5(11.4%)	0.37	0.01	3(6.8%)	2(4.5%)	8(18.2%)	0.31	0.039
Poorly	3(6.8%)	5(11.4%)	13(29.5%)			3(6.8%)	8(18.2%)	10(22.7%)		
Total	12(27.3%)	12(27.3%)	20(45.4%)			12(27.3%)	13(29.5%)	19(43.2%)		

The status of the portal invasion and intra-hepatic invasion of 44 patients with hepatocellular carcinoma were also listed in table 1. The correlation between Grp78 expression and the status of portal invasion and intra-hepatic invasion of 44 patients with HCC were analyzed by Spearman test. As shown in table 3, the expression levels of Grp78 were positively correlated with portal invasion and intra-hepatic invasion in 44 patients with HCC (r = 0.724, *P *= 0.00; r = 0.679, *P *= 0.00). The statistical analysis also revealed a positive correlation between Grp78 and FAK levels in 44 tissue samples of HCC (Table 4).

**Table 3:** Correlation between Grp78 level and the status of portal invasion and intrahepatic invasion in 44 patients with HCC.

	Portal invasion				Intrahepatic invasion			
Grp78 level	Y	N	r	*P*	Y	N	r	*P*
1	0(0.0%)	12(27.3%)			0(0.0%)	12(27.3%)		
2	4(9.1%)	8(18.2%)	0.724	<0.01	2(4.5%)	10(22.7%)	0.679	<0.01
3	17(38.6%)	3(6.8%)			15(34.1%)	5(11.4%)		

**Table 4:** Correlation between Grp78 and FAK expressions in 44 patients with HCC.

	FAK level				
Grp78 level	1	2	3	r	*P*
1	6(13.6%)	2(4.5%)	4(9.1%)		
2	2(4.5%)	6(13.6%)	4(9.1%)	0.482	0.001
3	0(0.0%)	5(11.4%)	15(34.1%)		

### Grp78 promoted the invasion of hepatocellular carcinoma cells

**Figure 2:**
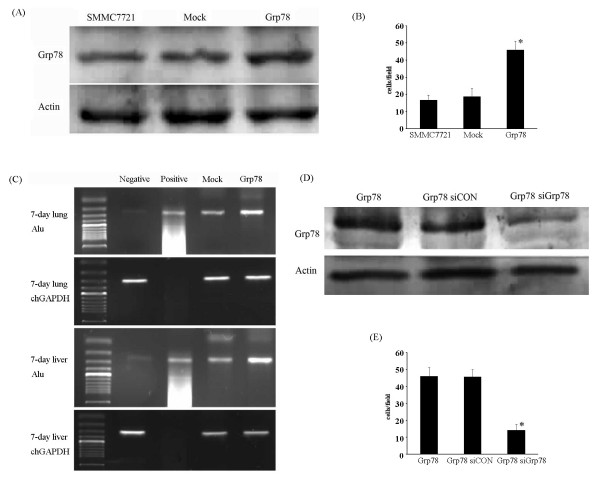
**Overexpression of Grp78 induces hepatocellular carcinoma cells invasion**. (A), Western blot of protein lysates from mock- and Grp78-transfected SMMC7721 cells, indicating Grp78 expression level. The lower blot was used as loading control. (B), A transwell assay using mcok- and Grp78-transfected cells was performed on inserts pre-coated with ECM gel. Images are representative of the invasion of Grp78 overexpressing cells in three separate experiments performed in triplicate. (C), A chick embryo metastasis assay was performed using mock- and Grp78-transfected cells suspended in PBS. The extent of tumor cell invasion was evaluated by human specific Alu PCR using DNA extracts from chick embryonic lungs and livers. Negative control: DNA extracts from untreated 7-day chick embryonic lungs and livers. Positive control: DNA extracts from human colorectal caricinoma. (D), Western blot analysis showing Grp78 level in Grp78 siRNA and control siRNA transfected Grp78 overexpressing cells. (E) Representative image of a transwell assay using Grp78 siRNA knockdown Grp78 overexpressing cells and control siRNA transfected Grp78 overexpressing cells for further elucidating the role of Grp78 in tumor cells invasion. The experiments were repeated for three times in triplicate.

To confirm our previous results, we transfected SMMC7721 cells with pcDNA 3.1(+)-Grp78 recombinant by Fugene HD followed by G418 selection. The transfection efficiency was assessed by western blot, indicating a significant increase (= 2.5 fold) as compared with mock-transfected cells (Fig.2A).

We examined the invasion of Grp78 overexpressing cells by transwell assay in vitro and chick embryo metastasis model in vivo respectively. Transwell assay revealed that SMMC7721 cells stably overexpressing Grp78 showed a significant increase in invasion (> 2 fold) as compared with mock-transfected cells (Fig. 2B). Chick embryo metastasis and Alu-PCR indicated a significant increase in invasion in Grp78 overexpressing cells (≈3 fold) over mock-transfected cells (Fig. 2C).

To confirm that the increased invasion of Grp78 transfected cells is caused by Grp78 itself, Grp78 level in Grp78 overexpressing cells was knockdown by siRNA transfection (Fig. 2D). We found that the increased invasion of Grp78 overexpressing cells was significantly inhibited (Fig. 2E). Taken together, these data suggested that overexpression of Grp78 promoted the invasion of hepatocellular carcinoma cells.

### Grp78 promoted cell spreading and cell polarity formation of hepatocellular carcinoma cells

**Figure 3:**
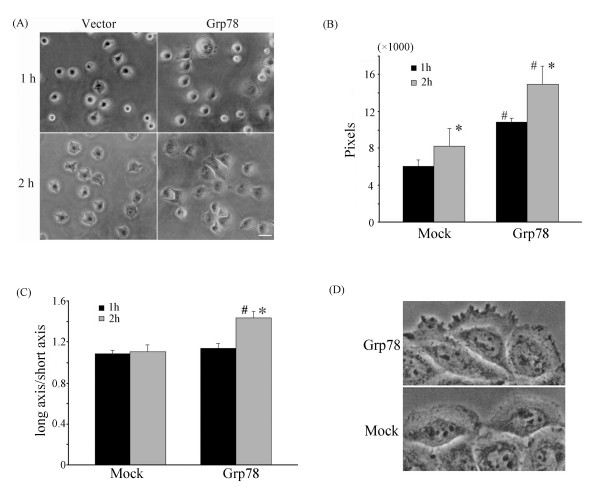
**Overexpression of Grp78 promotes the spreading and cell polarity formation of hepatocellular carcinoma cells**. (A), SMMC7721 cells that stably transfected with Grp78 or mock transfected were allowed to spread on Fibronectin (10 μg/ml) pre-coated surface for 2 hours. The extent of cell spreading was photographed under phase-contrast microscopy at 1 hour and 2 hours. This experiment was repeated for three times in triplicate. (Scale bar = 20 μm) (B), Overexpression of Grp78 in SMMC7721 cells promoted cell spreading which is represented as the mean area of cells (pixels). (C), Overexpression of Grp78 in SMMC7721 cells promotes cell polarity formation which is depicted as the ratio of long axis and short axis. (D) SMMC7721 cells that stably transfected with Grp78 form obvious thin, transparent lamellipodia at the wound edge compared with mock-transfected cells.

To determine if Grp78 affected the spreading of tumor cells, we observed the spreading status of Grp78 overexpressing cells for 2 h after the cells were replated on fibronectin-coated coverslips (10 μg/ml). We found that Grp78 overexpressing cells exhibited an accelerated spreading as compared with mock-transfected cells. Most Grp78 overexpressing cells exhibited a polar, elongated, morphology after 2 h of replating. However mock-transfected cells presented a round symmetric morphology without obvious polarity formation (fig 3A).

The status of cell spreading and cell polarity formation was analyzed using Osiris software (HUG). The extent of cell spreading was depicted as the mean area of tumor cells. We found that the mean area of Grp78 overexpressing cells was 14971 ± 1929 pixels after 2 h of replating on fibronectin -coated surface. By contrast, the mean area of mock-transfected cells was 8234 ± 1905 pixels, indicating that overexpression of Grp78 promoted the spreading of tumor cells (Fig. 3B).

The extent of cell polarity formation of Grp78 overexpressing cells was represented as the ratio of long axis and short axis. The results revealed that the mean value of Grp78 overexpressing cells was 1.5 ± 0.07, indicating an asymmetric, elongated morphology. The mean ratio of mock-transfected cells was 1.1 ± 0.06, suggesting a symmetric, round morphology (Fig.3C).

We also observed obvious lammellipodia formation in Grp78 overexpressing cells. Cell monolayer was carefully wounded by sterile pipette and the morphology of the cells at the wound edge was observed. Grp78 overexpressing cells formed obvious transparent membrane ruffles at leading edge, while the membrane ruffles was not obvious in mock-transfected cells (Fig. 3D).

### Overexpression of Grp78 enhanced the phosphorylation of FAK in hepatocellular carcinoma cells

**Figure 4:**
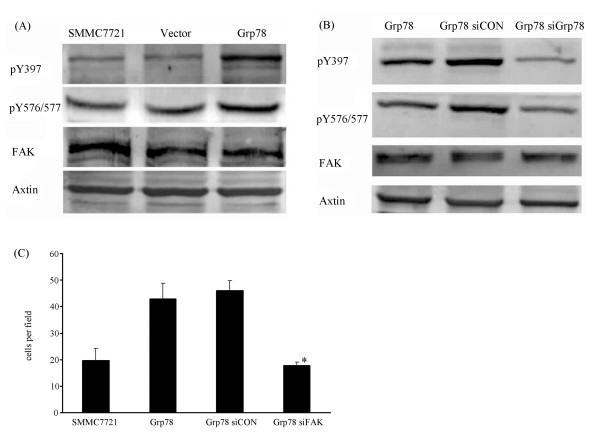
**Overexpression of Grp78 up-regulates FAK activation**. (A), Western blot of phosphorylated Tyr 397 and Tyr 576/577 from serum starved mock-transfected cells and Grp78-transfected cells plated on Fibronectin for 1 hour. Corresponding total FAK is shown in the lower blot. (B), Western blot of phosphorylated Tyr 397 and Tyr 576/577 from serum starved Grp78 siRNA and control siRNA transfected Grp78 overexpressing cells plated on Fibronectin for 1 hour. (C), Representative image of a transwell assay using FAK siRNA and control siRNA transfected Grp78 overexpressing cells for investigating the effect of FAK siRNA knockdown in Grp78 overexpressing cells on Grp78 mediated tumor cells invasion. The experiments were repeated for three times in triplicate.

FAK is known to be an important intracellular signaling molecule that regulates tumor cells invasion. It is known that phosphorylation of Y397 of FAK results in its activation. We therefore reasoned that if overexpression of Grp78 affected FAK activation. Grp78 overexpressing cells showed a significant increase in pY 397 level (>2 fold) over mock-transfected cells, suggesting that overexpression of Grp78 induced FAK activation (Fig. 4A). We also found that the expression of FAK in Grp78 overexpressing cells was equal to mock-trandfected cells.

Phosphorylation of Y576/577 is essential for maximal activity of FAK. We next examined pY576/577 level of FAK in Grp78 overexpressing cells. As compared with mock-transfected cells, a significant increase of pY576/577 level (>2 fold) was found in Grp78 overexpressing cells, indicating that overexpression of Grp78 promoted the maximal activity of FAK (Fig. 4A).

To confirm the role of Grp78 in the activation and activity of FAK, Grp78 level in Grp78 overexpressing cells was knockdown by siRNA transfection, pY397 and p Y576/577 levels were examined. We found that Grp78 siRNA knockdown in Grp78 overexpressing cells significantly decreased pY397 and p Y576/577 levels of FAK, indicating that the enhanced activation and activity of FAK in Grp78 overexpressing cells was caused by Grp78 itself (Fig. 4B).

To further elucidate the role of FAK in Grp78 mediated tumor cells invasion, we knockdown FAK in Grp78 overexpressing cells and examined if FAK knockdown affected the invasion of Grp78 overexpressing cells. We found that the increased invasion of Grp78 overexpressing cells was reversed to the level of mock-transfected cells, suggesting that Grp78 regulated tumor cells invasion in a FAK-dependent manner (Fig. 4C).

### Overexpression of Grp78 negatively regulates Rock activity

**Figure 5:**
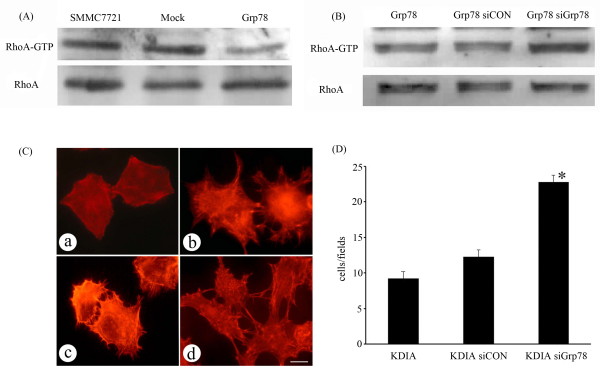
**Overexpression of Grp78 decreases Rock activity in hepatocellular carcinoma cells**. (A), lysates from mock- and Grp78 transfected cells were precipitated using Rhotekin-RBD bound sepharose beads and blotted with anti-RhoA. Corresponding total RhoA is shown in lower blot. (B), A GST pulldown assay using lysates from Grp78 siRNA and control siRNA transfected Grp78 overexpressing cells, indicating RhoA-GTP level. (C), Grp78 knockdown in Rock dominant negative recombinant (KDIA) transfected cells caused obvious stress fiber formation, indicating endogenous Rock activation. (Scale bar = 10 μm). (a), mock-transfected cells.(b) KDIA transfected cells. (c) Control siRNA transfected KDIA cells. (d), Grp78 siRNA knockdown KDIA cells. (D), Representative image of a transwell assay using Grp78 siRNA and control siRNA transfected KDIA cells, indicating Grp78 knockdown rescued the invasion of KDIA cells. The experiments were repeated for three times in triplicate.

Rho dependent kinase (Rock), a downstream Rho effector, plays critical roles in the regulation of tumor invasion. Overactivation of Rock restrains tumor invasion and metastasis by promoting stress fiber formation and stiffening the cell cortex [19,20]. We therefore determined if overexpression of Grp78 affected RhoA activity. Grp78 overexpressing cells showed a significant reduction of RhoA-GTP level (>2 fold) as compared with mock-transfected cells, suggesting that overexpression of Grp78 inhibited RhoA activity (fig 5A).

To validate the inhibitory effect of Grp78 on RhoA activity, we down-regulated Grp78 expression in Grp78 overexpressing cells by siRNA transfection. Interestingly, Grp78 siRNA knockdown in Grp78 overexpressing cells resulted in a significant increase (≈2.3 fold) in RhoA -GTP level (Fig 5B).

To further validate the effect of Grp78 on Rock activity, we down-regulated Grp78 level in cells stably expressing KDIA, a Rock dominant negative recombinant which inactivates endogenous Rock activity [21,22]. Grp78 siRNA knockdown KDIA transfected cells formed obvious stress fibers in cell cortex. By contrast, actin bundles distributed mainly in the periphery of KDIA transfected cells (fig 5C). We also found that Grp78 siRNA knockdown in KDIA transfected cells rescued the invasion defect phenotype, suggesting that Grp78 knockdown activated endogenous Rock activity (Fig. 5D).

### Overexpression of Grp78 promotes p190RhoGAP phosphorylation in a FAK-dependent manner

**Figure 6:**
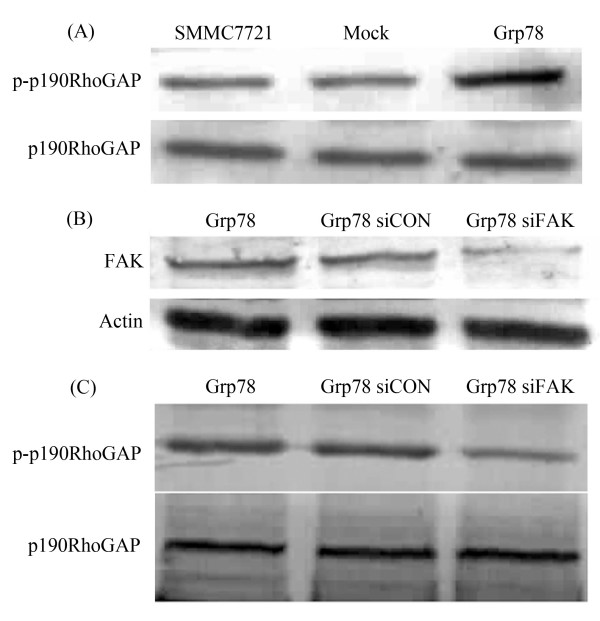
**Overexpression of Grp78 promotes p190RhoGAP phosphorylation in a FAK-dependent manner**. (A) Overexpression of Grp78 in hepatocellular carcinoma cells enhanced the phosphorylation of p190RhoGAP. (B) Transfection of FAK specific siRNA decreased FAK levels in Grp78 overexpressing cells. (C) Western blot analysis of phospho-p190RhoGAP levels from FAK siRNA and control siRNA transfected Grp78 overexpressing cells.

Previously, we have demonstrated that Grp78 enhanced FAK phosphorylation and inhibited RhoA activity in hepatocelluar carcinoma cells. We further reasoned how Grp78 regulated Rock kinase activity. We found a significant increase of phospho-p190RhoGAP level in Grp78 overexpressing cells, suggesting that Overexpression of Grp78 enhanced the phosphorylation of p190RhoGAP (Fig. 6A). To explore if Grp78 enhanced the phosphorylation of p190RhoGAP in a FAK dependent manner, FAK level in Grp78 overexpressing cells was down-regulated by siRNA transfection, the phosphorylation status of p190RhoGAP was determined (Fig. 6B). We found a significant reduction of phospho-p190RhoGAP level in FAK siRNA knockdown Grp78 overexpressing cells as compared with mock-transfected cells, suggesting that Grp78 promoted p190RhoGAP phosphorylation in a FAK dependent manner (Fig. 6C).

## Discussion

Grp78 is a stress-inducible protein which is ubiquitously expressed in animal cells. Compared with normal tissue, the expression of Grp78 in many human cancers is strongly up-regulated. Grp78 is implicated in the oncogenesis, progression, drug resistance of cancer cells. Although the emerging importance of Grp78 in tumor progression is well recognized in many human cancers, the data in hepatocellular carcinoma is still fewer. We found a negative correlation between Grp78 level and the portal invasion and intra-hepatic invasion in patients with hepatocellular carcinoma, suggesting that Grp78 is involved in the invasion of hepatocellular carcinoma. This conlusion was in agreement with the data in other human cancers.

Invasion of cancer cells to adjacent tissues is an essential characteristic of tumor progression which is tightly regulated by many signaling molecules. Herein, we found that overexpression of Grp78 in hepatocellular carcinoma cells caused a two to three fold increase in the invasion capability. Moreover, Grp78 siRNA knockdown in Grp78 overexpressing cells reversed the higher invasion capability near the level of control cells. Previous studies have linked Grp78 knockdown to the decreased invasion capability of cancer cells in breast cancer, gastric cancer and prostate cancer. Our results revealed that Grp78 overexpression induced the invasion of hepatocellular carcinoma.

Although the inhibitory roles of Grp78 knockdown in tumor invasion have been reported in many human cancers, the molecular mechanism remains obscure. These observations raised an issue. How Grp78 might induce the invasion of cancer cells? Our results preliminarily demonstrated that two major mechanisms are involved in the increased invasion mediated by Grp78: enhancement of FAK activation, inhibition of RhoA activity.

FAK is a key member of integrin-mediated signaling pathways and plays critical roles in the adhesion, invasion and migration [23]. Increased activation of FAK is associated with more invasive and aggressive phenotypes [24]. We found that overexpression of Grp78 in hepatocellular carcinoma cells increased FAK pY397 level suggesting that Grp78 may be involved in the regulation of FAK activation. This result was confirmed by the fact that Grp78 siRNA knockdown in Grp78 overexpressing cells caused a significant decreased FAK activation. We also found that FAK siRNA knockdown in Grp78 overexpressing cells partially inhibited the increased invasive capabilities caused by Grp78, suggesting that FAK is involved in Grp78 mediated tumor invasion. It is noteworthy that the expression of FAK was unchanged in Grp78 overexpressing cells compared with mock transfected cells, suggesting the promotion role of Grp78 on FAK activation was mainly by modulating the phosphorylation of FAK.

Basal level RhoA activity is essential for the invasion and metastasis of cancer cells. However, overactivation of RhoA inhibits this process [25]. Many data have revealed that RhoA is a downstream effector of FAK which negative regulates the activity of RhoA. We found that RhoA-GTP level in Grp78 overexpressing cells is relatively lower than mock transfected cells. Moreover, Grp78 siRNA knockdown in Grp78 overexpressing cells significantly increased RhoA-GTP level, suggesting that Grp78 may negatively regulate RhoA activity. This conclusion is further confirmed by Grp78 siRNA knockdown in Rock kinase dead cells which is transfected with KDIA, a Rock dominant negative recombinant. We observed obvious stress fiber formation in Grp78 siRNA knockdown KDIA transfected cells and partially rescue of the invasion ability, suggesting that overexpression of Grp78 activated endogenous Rock. This conclusion is in agreement with the previous studies that FAK is a negative regulator of RhoA activity in many human cancers.

Although the functional connection between FAK and RhoA has been reported in many human cancers, how Grp78 inhibited Rock activity in hepatocellular carcinoma should be elucidated. We found that the RhoA-GTP level in FAK siRNA knockdown Grp78 overexpressing cells is significantly higher than in Grp78 overexpressing cells, suggesting that Grp78 promoted RhoA activity by a FAK dependent manner.

p190RhoGAP is a member of GTPase activating proteins which stimulate the GTPase activity on RhoGTPase and hydrolyzed RhoA-GTP [15]. We observed that increased phosphorylation level of p190RhoGAP in Grp78 overexpressing cells. However, FAK siRNA knockdown in Grp78 decreased the phosphorylation level of p190RhoGAP. These data suggested that p190GAP may play important role in the connection between FAK and RhoA in Grp78 mediated tumor invasion.

## Conclusion

Taken together, Grp78 promoted the invasion of hepatocellular carcinoma both in vitro and in vivo. Overexpression of Grp78 in hepatocellular carcinoma cells enhanced the activation and activity of FAK which negatively regulated Rock kinase activity by promoting the phosphorylation of p190RhoGAP.

## Competing interests

The authors declare that they have no competing interests.

## Authors' contributions

Rongjian Su designed this research and drafted the manuscript. Zhen Li, Hongdan Li, Cuifen Bao and Jia Wei performed the experiments. Liufang Cheng collected the clinicopathological data of 44 patients with HCC and analyzed these data. All the authors read and approved the final manuscript.

## Pre-publication history

The pre-publication history for this paper can be accessed here:

http://www.biomedcentral.com/1471-2407/10/20/prepub
